# Understanding and Modulating Antibody Fine Specificity: Lessons from Combinatorial Biology

**DOI:** 10.3390/antib11030048

**Published:** 2022-07-14

**Authors:** Gertrudis Rojas

**Affiliations:** Protein Engineering Department, Center of Molecular Immunology, Havana CP 11300, Cuba; grojas@cim.sld.cu

**Keywords:** affinity maturation, antibody engineering, epitope mapping, libraries, mutagenesis, paratope, peptide mimotopes, phage display, yeast display

## Abstract

Combinatorial biology methods such as phage and yeast display, suitable for the generation and screening of huge numbers of protein fragments and mutated variants, have been useful when dissecting the molecular details of the interactions between antibodies and their target antigens (mainly those of protein nature). The relevance of these studies goes far beyond the mere description of binding interfaces, as the information obtained has implications for the understanding of the chemistry of antibody–antigen binding reactions and the biological effects of antibodies. Further modification of the interactions through combinatorial methods to manipulate the key properties of antibodies (affinity and fine specificity) can result in the emergence of novel research tools and optimized therapeutics.

## 1. Introduction

Antibodies form the largest family of binding proteins, able to recognize virtually every kind of target (called antigens), ranging from small organic compounds known as haptens [1,2] to complex molecules such as proteins, carbohydrates, lipids, nucleic acids, and their combinations. This functional versatility is dictated by the extreme diversity of antibody molecules, which combine two different polypeptides (heavy and light chains), each having a particular variable region. Although variable regions share a similar global architecture, they include three protruding hypervariable loops, characterized by even larger primary amino acid (aa) sequence variability, as well as by length and conformational diversity. In functional terms, these loops are the complementarity determining regions (CDRs). The spatial array formed by the six CDRs of each antibody (three from the heavy and three from the light chain) shape a unique binding site (or paratope), able to establish an interaction network with target antigen(s) [3].

The counterpart of an antibody paratope is the antigen portion directly involved in recognition, known as epitope. As early as in 1897, Paul Ehrlich described the capacity of antibodies to neutralize toxins in the following terms: ‘‘…the ability of toxins to bind antibody must be due to a specific atom group of the toxin complex, which shows a maximum specific relationship to an atom group of the antitoxin …’’ [4]. A large antigen thus contains multiple epitopes (atom groups according to Ehrlich), as many as different antibodies could bind it. Methods such as x-ray diffraction are useful to elucidate the structure of antibody–antigen complexes, providing pictures of the interface between both [5]. On the other hand, functional methods aimed at determining the contribution of each chemical group to binding delineate an energetic landscape of the interaction beyond describing the mere proximity of atom groups [6].

All antibodies, and many of the antigens they recognize, are proteins. Paratopes are formed by peptide loops, and epitopes are often groups of neighbor solvent-exposed aa side chains on protein surfaces (in the case of antigens of peptide nature). Therefore, functional exploration of paratope–epitope interface is frequently equivalent to dissecting the role of individual amino acids and their combinations in binding. Combinatorial biology methods, allowing the quick biosynthesis and screening of diverse collections of peptides and proteins, are ideal tools for that. Such approaches, mainly represented by phage [7] and yeast display [8], have been successfully used to determine critical residues within paratopes and epitopes. Another field of application, beyond understanding pre-existing interactions, is their optimization/modification through directed evolution to generate new paratopes with the desired properties.

The current review summarizes the experiences of our group and other teams in the study and manipulation of antibody–antigen interactions though combinatorial biology. Even though multiple structural, functional, and theoretical (in silico modeling) approaches have indeed contributed to these general goals and diverse techniques are mentioned along the text, this article is focused on a subset of them, based on display technologies such as phage and yeast display. Considerations about the usefulness and limitations of particular methods within this field are included, and their potential applications are discussed. Special emphasis is made on the implications of findings derived from combinatorial biology approaches for our understanding of antibody chemistry and biology.

## 2. The Relevance of Functional Epitope Mapping for Immunologists and Antibody Engineers

The process of determining the location and chemical nature of each epitope is known as epitope mapping. Although the review will focus on antigenic determinants recognized by antibodies, the reader should know that the terms ‘epitope’ and ‘epitope mapping’ are not restricted to antigen–antibody interactions but can be used in the context of peptide antigen recognition by T cell receptors and even of other biomolecular noncovalent interactions outside the immunology field. Epitope mapping procedures can be classified in two main categories: structural and functional. Structural approaches determine those chemical groups in the antigen that are in close proximity to the antibody (and are thus likely to interact with it), through the study of crystal structures of antigen–antibody complexes and other techniques. Functional methods dissect the direct roles of individual antigen moieties in recognition, and the hierarchy of their energetic contributions. Even though the molecular nature of antigens and their epitopes can be broadly diverse (see the previous section), proteins represent an important antigen class due to their abundance and relevance for immune responses. Functional epitope mapping in the case of protein targets is aimed at underscoring the roles of amino acids (and groups of them) in recognition, describing the subset of residues directly engaged in the interaction and their relative contributions to binding energy. The current review is mainly focused on the study of this kind of antigen.

The importance of functional epitope mapping is supported by several reasons. First of all, fine details of epitope recognition determine the biological effects of antibodies. Even subtle differences in specificity, such as the ones reported for antibodies neutralizing the interaction between Interleukin-2 and its multi-chain receptor, can result in totally different outcomes (enhancement versus downmodulation of immune responses) [9,10,11]. For membrane antigens, the precise orientation of the antigen–antibody complex with respect to the cell membrane (determined by the bound epitope) has a big influence on immunological effector functions, as it has been described for anti-CD20 antibodies against partially overlapping epitopes with different effects on malignant cells [12].

A view from the industry reveals that the availability of large engineered antibody repertoires (libraries) provides the opportunity to discover dozens and even hundreds of antibodies against each antigen [13,14]. Such capacity contrasts with the bottlenecks at product development and clinical trials. There is an urgent need to characterize antibody fine specificities since the beginning and choose the ideal ones for a given application. The requirement to obtain patent protection upon antibody discovery is an additional motivation to go deeper into functional mapping. Epitope specificity can be a source of novelty and non-obviousness for new antibodies. The number of patent applications including claims related to epitope description is increasing, but they are subject to substantial scrutiny to find genuine distinctive features [15]. Epitope mapping should be performed at the highest possible resolution (at amino acid and even atomic level) in order to formulate suitable claims.

A final reason to perform detailed functional epitope mapping refers to the changing nature of many antigens. Even minor changes within the epitope can abrogate antibody recognition. Monoclonal antibody (mAb) therapy can result in the emergence of target variants that have lost the original epitope and are subsequently mAb-resistant. This phenomenon was described in patients who stopped responding to treatment with cetuximab (an antibody targeting the epidermal growth factor receptor (EGF-R) overexpressed in tumors), due to a single mutation in the EGF-R extracellular domain [16]. Further treatment with a second antibody (panitumumab) recognizing an overlapping, but not identical, epitope on the same target [17] restored therapeutic responsiveness. Other tumor target-associated mutations have been described [18,19,20]. Something similar happens during infectious diseases, as exemplified recently by the emergence of mutated SARS-CoV-2 variants nonrecognized by antiviral neutralizing antibodies [21,22]. Anticipated knowledge of the effects of epitope mutations on antibody binding, and the availability of several antibodies with divergent fine specificities against the same target could help to overcome therapeutic resistance related to epitope evolution.

## 3. Screening Reactivity of Antibodies against Antigen Fragments: The Simplest Way to Locate Epitopes

The most obvious approach to determine the residues involved in antigen recognition by a given antibody is the screening of small pieces of the antigen (short peptides representing segments of its polypeptide sequence). Some of the oldest and most exploited epitope mapping approaches are based on chemically synthesized peptides [23,24]. Reactivity of one or more fragment(s) with the antibody indicates the epitope location. An alternative to synthetic chemistry is the production of antigen fragments by expressing gene segments in recombinant host cells. The widespread use of genetic engineering makes this approach feasible at any molecular biology laboratory. Fragment length is not a limiting factor for biosynthesis, as either small segments corresponding to minimal epitopes or larger fragments able to fold and recapitulate discontinuous epitopes formed by residues distant in the primary sequence but close in the 3D structure can be produced in that way.

Biosynthetic approaches went far beyond classical screening of recombinant proteins. Combinatorial biology emerged as a very efficient way to manipulate collections of antigen fragments. There are several techniques to obtain selectable particles (viruses or cells) displaying a single protein fragment each and containing its coding gene. The oldest and most extended display platform is phage display, awarded with the Nobel Prize in Chemistry in 2018 [7,25]. The pioneering work of G.P. Smith was indeed the first example of antigen fragments’ display [26]. He displayed an enzyme fragment on filamentous phages and showed that an antibody against the enzyme was able to capture these phages within a complex mixture. He concluded that ‘‘fusion phage may provide a simple way of cloning a gene when an antibody against the product of this gene is available’’.

**Figure 1:**
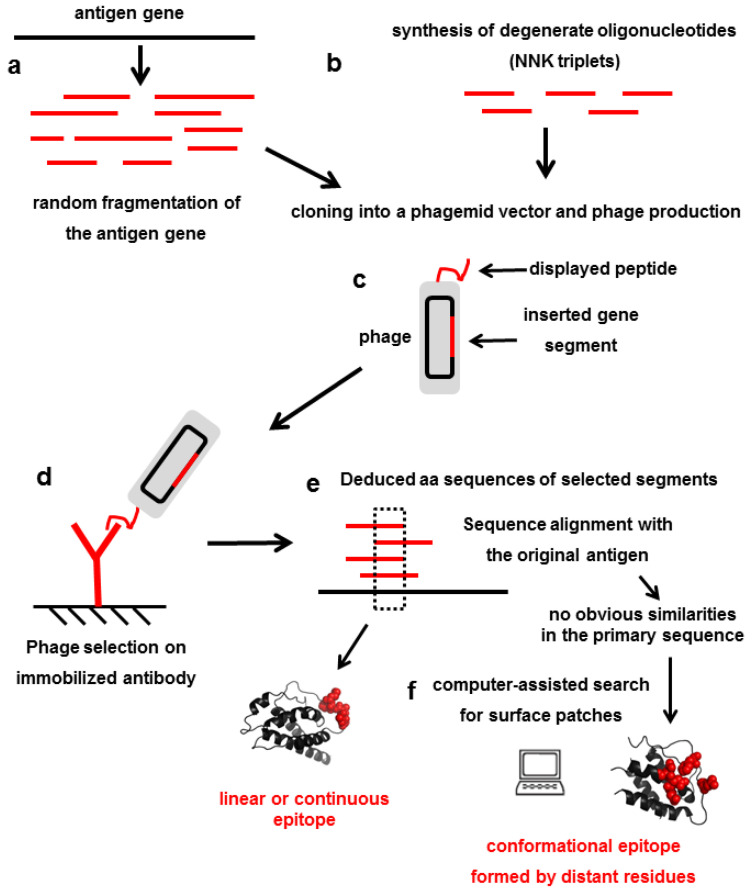
Epitope identification using libraries of antigen fragments or random peptides. (**a**) Generation of fragments from the antigen gene. (**b**) Synthesis of short segments of DNA encoding random peptides. (**c**) Cloning of antigen gene-derived/random DNA segments in suitable phagemid vectors and production of phages displaying the corresponding peptides. (**d**) Selection of phages displaying peptides that are recognized by the antibody under investigation. (**e**) Sequencing of the inserted DNA fragments from selected phagemids, deduction of peptide sequences, and alignment with the whole protein sequence of the antigen, allow direct identification of the antigen segment that is recognized (linear or continuous epitope). (**f**) If there is no obvious similarity between the isolated peptide ligands and the antigen, peptides can resemble a cluster of amino acids in the antigen 3D structure. In these cases, the conformational or discontinuous epitope is located through computer-assisted exploration of the antigen surface.

This experience was extended to the display of antigen fragments to locate the epitope(s) within them. Large fragment phage display led to successful recapitulation of conformational epitopes of HER-2 (a complex antigen having four extracellular domains and multiple disulfide bonds) [27]. Increasing the scale allowed panning large libraries of phage particles (displaying up to more than 10^10^ unique antigen fragments) on an antibody-coated surface. Only those phages displaying the segment(s) recognized by the antibody are captured. Eluting bound phages and sequencing their DNA allows the definition of the minimal epitope for the selector antibody, from the analysis of the overlapping sequence shared between encoded antigen fragments [28,29]. Figure 1 shows the workflow for constructing (a) and screening (c–e) phage-displayed libraries of antigen fragments.

Mapping strategies based on antigen fragments’ display on other (nonfilamentous) bacteriophages have been developed [30]. Alternative combinatorial display technologies are also available. Antigen-displaying selectable particles are not only viruses. If the genes coding for antigen fragments are fused to the gene corresponding to one surface protein and cloned into suitable expression vectors, both bacteria and yeast cells can display these fragments on their surface. Specific staining with an antibody and isolation by fluorescence-activated cell sorting (FACS) allow picking those cells that display the recognized antigen fragment(s) [31,32]. As each cell displays a single antigen fragment and contains the corresponding genetic information, DNA sequencing can then be used to define the epitope identity.

Biological libraries of antigen fragments have two major advantages over synthetic peptide collections. The first one is diversity. It is easier to obtain millions (and even thousands of millions) of fragments by parallel gene cloning in expression vectors than by chemical synthesis. Large library sizes rule out the need for a precise a priori definition of fragment length and composition. Random fragmentation of antigen genes renders a wide range of DNA segments´ lengths. The minimal epitope is likely to be contained in multiple fragments within the library (with variable degrees of overlapping). Alignment of the broad variety of fragments picked by a selector antibody thus results in a highly reliable epitope definition. Additionally, some epitopes are better reproduced in a biosynthetic environment (bacterial periplasm or yeast endoplasmic reticulum) where disulfide bonds can be formed and the antigen fragments´ folding resembles native antigen conformation.

## 4. Mimicking Epitopes with Short Random peptides: An Indirect Approach for Epitope Mapping

Mapping antibodies against different antigens requires the construction of multiple antigen fragments´ libraries. Universal libraries displaying random amino acid sequences have been envisaged as single-pot reservoirs of unlimited antigenic diversity of general usefulness, from which short peptides resembling any particular epitope could be selected. Cloning a stretch of degenerate triplets that code for the mixture of the 20 amino acids (such as NNK) in a suitable vector is enough to obtain these libraries, which have been used during decades for epitope mapping [33,34]. The number of library clones (each displaying a unique peptide) frequently reaches up to 10^9^–10^10^. This upper limit of library sizes is dictated by the ability to transform host cells with foreign genetic constructs using electroporation (a highly efficient transformation method). Random peptides may differ in length (from a few amino acids up to 30-mers). Only libraries containing seven to eight random residues or fewer can reach a complete coverage of all possible aa combinations within the peptide pool. Theoretical combinatorial diversity ranges from 1.28 × 10^9^ in the case of heptapeptides to 2.56 × 10^10^ octapeptides. Libraries of longer peptides provide a vast diversity of molecules to be screened but do not guarantee full coverage of all potential aa sequences. Anyway, as the interactions with an antibody often involve just a few residues within the peptide sequence, the use of long peptides can be envisaged as a strategy to increase diversity beyond the library size measured as the number of clones. A single clone displaying a long peptide actually presents to the antibody multiple in-tandem or overlapping peptide motifs.

As short peptides are considered too flexible to strongly interact with the selector antibody, disulfide-constrained libraries have been explored as sources of more ‘rigid’ and potentially higher-affinity peptides [35]. In the so called ‘cyclic’ libraries, a random sequence is flanked by two cysteines which form a disulfide bridge constraining its conformational flexibility. Despite the theoretical attractiveness of ‘cyclic’ library design, conventional ‘linear’ libraries including Cys within its random composition also render Cys-constrained peptides if they are actually better binders to the selector antibody. Isolation of peptide motifs including two Cys residues separated by a constant number of amino acids is frequent. In these cases, formation of disulfide bonds is usually required for reactivity [36]. Sometimes such Cys-constrained loops in the peptide(s) reproduce natural loops in the antigen, as it was reported for an immunodominant Cys-flanked epitope of gp41 HIV antigen [37]. While in the ‘cyclic’ libraries, the inter-Cys distance is determined a priori, libraries containing a single fixed Cys residue allow us to select a second Cys at other positions, forming the ideal disulfide-bridged loop for binding [38]. Besides the presence of fixed Cys, either paired or unpaired, peptide composition can be totally random or biased by design. Rational design and the use of controlled trinucleotide mixes for library construction can result in the equimolar representation of every aa at each position, the exclusion of certain structurally singular residues such as Cys or Pro from diversified positions, or any other desired feature.

The identification of peptide sets recognized by a given antibody is used to define common motifs likely to represent the minimal epitope. Alignment of these motifs with the original antigen sequence reveals the precise epitope location. However, epitope assignment is complicated by the following reasons: similar amino acids can replace the original epitope residues in selected peptides, and not all residues within an antigen segment interact with the paratope. Therefore, inspection of selected peptides should not be limited to the search for an exactly matching segment within the primary sequence of the antigen, but often requires the identification of sequence motifs sharing physicochemical properties. A consensus motif might be interrupted by positions occupied by any amino acid or by several residues without any obvious similarity, neither between them nor with the original antigen segment. Characterization of thousands of epitope-resembling peptides has revealed a wide diversity of epitope fingerprints in terms of the number, spacing, and relevant physicochemical properties of crucial residues.

Further complexity is added by the fact that library screening does not always render a clear consensus motif. A single peptide might dominate selection due to an intrinsic advantage not necessarily related to affinity for the selector antibody, for instance enhanced display on the phage surface. The isolation of a single peptide precludes the identification of the critical residues due to the presence of additional irrelevant amino acids along the same peptide. Alternatively, multiple different peptides without any distinguishable shared sequence feature could be selected by a given antibody. Target-unrelated peptides (TUPs) can be picked due to their ‘sticky’ nature, or to the ability to bind the solid surface, the blocking agents, or other molecules involved in the selection procedure [39]. The Fc region of the selector antibody could select Fc-binding peptides not interacting with the paratope. Known TUPs can be excluded after sequencing [40,41], but more importantly, panning should be optimized to avoid nonspecific interactions and restrict selection to paratope binders.

Much more challenging is the fact that peptides totally unrelated to the epitope can be selected by the paratope itself [42]. As the paratope is formed by six hypervariable loops, only some loops (and some residues within them) are actually in contact with the epitope and could interact with peptides in a similar fashion. The remaining residues/loops could establish a totally different network of interactions with peptides that do not resemble the epitope at all. One of the most stringent tests to determine whether peptides actually reproduce a portion of the antigen is the induction of antibodies sharing the original antigen specificity with the selector antibody, by immunization of animals with the peptides [43]. That would be a definitive indication of true antigen mimicry or its absence. The ability of a given paratope to accommodate totally different peptide ligands has important implications. It reflects the potential of a single paratope to react with more than one epitope, even derived from structurally unrelated antigens. This notion challenges the classical view of antibodies as strictly monospecific binding molecules, and provides a rational explanation for the appearance of unexpected cross-reactivities of therapeutic antibodies against nontargeted antigens in the body [44]. At the same time, such a knowledge opened new avenues in antibody engineering, such as the creation of paratopes with dual specificity able to bind two targets with high affinity and specificity (see Section 10).

If an antibody recognizes a conformation-sensitive epitope present in the folded protein antigen, but not in its denatured version, it is very likely that the selected peptide(s) do not reproduce any primary antigen sequence segment, but recapitulate 3D spatial arrays of the same (or similar) amino acids. These peptides are called mimotopes. Matching mimotopes and surface patches is even more complex than sequence alignment of linear epitopes. This process can be assisted by diverse computational tools. Improvement of such in silico methods has been as relevant as the evolution of experimental mapping techniques. Some of these tools are listed here: Mapitope [45], MIMOX [46], MIMOP [47], PepSurf [48], Episearch [49], LocaPep [50], MimoPro [51], GuiTope [52], Pep-3D-Search [53], and 3-D epitope explorer [54]. Figure 1 shows the workflow for phage-displayed random peptide library construction (b) and screening (c–f).

Peptide mimotopes can be selected by antibodies raised against nonprotein antigens such as carbohydrates, lipids and small organic compounds [36,38]. In these cases, mimicry cannot be due to the presence of identical chemical moieties in the epitope and its mimotopes. Antigenic mimicry can be limited to the ability of a peptide ligand to accommodate in the same paratope that recognizes a nonprotein epitope through an unrelated network of interactions, as previously discussed. Alternatively, a peptide mimotope may have the capacity to recapitulate the interactions with the same side chains of the paratope that are involved in binding to the original nonprotein antigen. When peptide ligands are true mimics of biologically relevant epitope(s), being them either protein or nonprotein in nature, they are able to elicit antibodies with the same specificity as the original one. This property is called immunogenic mimicry, and has been exploited to reproduce valuable properties of known monoclonal antibodies or antisera by immunization with suitable mimotopes, which can act as surrogate antigens [55,56].

## 5. Functional Epitope Mapping by Comprehensive Mutagenesis Scanning of Antigen Surface

Antigen fragments and peptide mimotopes can differ from the actual antigen. Even if all the critical residues are contained within a given fragment, their relative orientation in a short peptide might deviate from the one in the folded antigen. As the antibody ‘sees’ a spatial arrangement of atoms, this can preclude recognition. An alternative to antigen fragmentation is identifying the critical residues in the context of the whole almost-native antigen by introducing epitope-disrupting mutations. Site-directed mutagenesis scanning of the antigen is thus the method of choice for fine functional epitope mapping [57]. Combinatorial biology methods have come to make possible high throughput generation and screening of huge numbers of mutated antigen variants. Successful examples are based on a wide variety of techniques, differing in the display platform used, the degree of variability introduced in the antigen sequence, and the methods chosen for diversification, selection and analysis of antigen variants.

**Figure 2:**
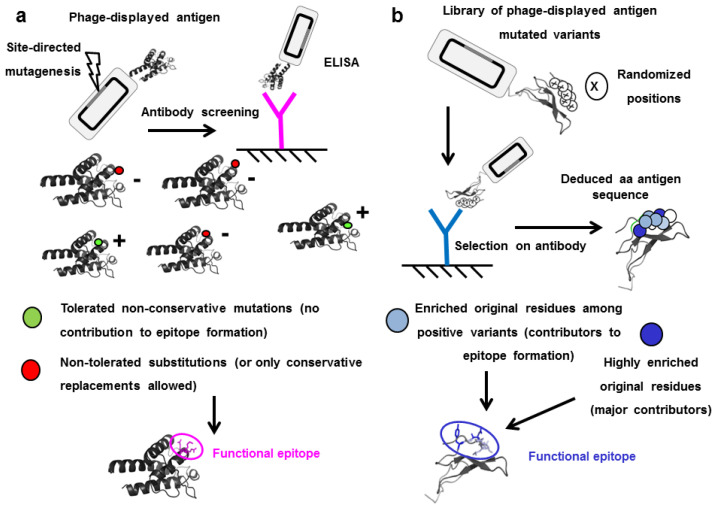
Epitope identification through mutagenesis scanning of the antigen surface. (**a**) Phage-displayed antigen is diversified through site-directed mutagenesis targeting a candidate antigenic area previously identified. Single mutated variants are tested by ELISA with the antibody under investigation, and classified as positive or negative. Those positions that cannot be mutated without abolishing antigen recognition (or where only conservative replacements are accepted) are defined as functional contributors to the epitope. (**b**) Combinatorial mutagenesis within a candidate antigenic region results in construction of a library of multiple mutated antigen variants. Phage panning on an immobilized antibody leads to selection of recognized (positive) variants. Sequencing reveals that several original residues are enriched to different extents among them, and the whole cluster is thus identified as the functional epitope.

Many antigens (or antigen domains) can be displayed on filamentous phages in a properly folded and fully antigenic form [58]. The combination of simple mutagenesis methods such as Kunkel reaction with phage ELISA is powerful enough to detect those residues that cannot be mutated without disturbing antigenicity [59]. Once one of such residues is identified, a comprehensive scanning of all positions within a given radius of this starting point (usually 8–12Å) can be performed. Although replacements by alanine (Ala scanning) can be used [60], randomization of every position under study has a much greater potential. Substitutions by Ala only show the importance of the original residue when it establishes highly critical interactions through its side-chain that cannot be recapitulated at all by Ala. Replacement by the mixture of the other 19 aa, on the other hand, can reveal additional information about residues that are not so critical, but cannot be replaced by any amino acid due to their proximity to the functional epitope and/or their partial involvement in epitope formation. Nontolerated mutations thus reveal which antigen positions are critical (or just influential) for antibody recognition, while the pattern of tolerated replacements underscores the precise amino acid properties (hydrophobicity, polarity, charge, shape, size, particular chemical moieties) that are required for, or compatible with, binding. The approach, illustrated in Figure 2a, is thus useful to establish not only the epitope location but also its detailed physico-chemical landscape [10,11,61]. Gain-of-recognition experiments are very powerful as confirmatory assays. Once the critical residues are identified, their introduction into the scaffold of a similar nonrecognized antigen (for instance, a homologous protein from another species) can be used to recapitulate the original epitope, as definitive proof of its identity [10,62].

Identification of epitopes by site-directed mutagenesis usually depends upon the existence of individually critical residues within the antigen, which cannot be changed without abolishing or reducing recognition. Many epitopes include at least one of such crucial amino acids. However, the antibody does not ‘see’ isolated amino acids. Binding is the net result of a network of interactions between two groups of side chains (from the paratope and from the epitope) at the molecular interface. The combination of all these interactions, including salt bridges, hydrogen bonds, hydrophobic interactions and van der Waals forces, determines binding. Some of them are rather weak, and their influence is only evident in the context of extensive additive and/or cooperative effects mediating recognition of the whole epitope. The energetic landscape of some epitopes is thus diffuse, with no individually critical residues. The relevance of the whole cluster of residues can become evident in two ways, after combinatorial mutagenesis of a given surface patch within a phage library [63]. The first is loss-of-recognition of most mutated molecules of the library, which confirms epitope location. The second is a gain-of-recognition approach, as phage affinity selection from this library (containing mainly negative antigen variants) can underscore a set of antibody-recognized variants which tend to keep the original residues (or functionally equivalent aa) at some positions. These data provide information about residues in the cluster that are likely to contribute to binding in the context of the whole epitope (Figure 2b).

Simultaneous randomization of several neighbor positions in the 3D structure of the antigen can be considered a version of random peptide libraries. Chemical diversity of side chains is equally broad, but when the original antigen is used as the scaffold, it constrains the spatial disposition of the random residues and forces them to adopt a conformation compatible with antigen folding. The antigenic universe in the library is thus restricted to a structural space closer to the original antigen. Clusters of residues selected in that way can be considered mimotopes but are less likely to be irrelevant mimotopes not related to the original epitope (compared with free random peptides).

Yeast display—based in the presence of a single-antigen mutated variant anchored to the membrane of each recombinant yeast cell and the ability to label antibody-reactive cells, sort them by FACS and sequence the inserted antigen genes—was one of the first combinatorial platforms used for epitope mapping [64]. These libraries made use of the introduction of random mutations along the antigen gene through error-prone polymerase chain reaction (PCR). The completeness and accuracy of functional epitope maps thus generated were limited by the fact that only a few randomly generated mutations target residues around the actual epitope, leaving important positions unexplored. The presence of more than one mutation in the same variant complicates the interpretation of results, and undesired random targeting of residues with a crucial importance in global antigen folding (Cys, Pro or amino acids forming the protein hydrophobic core) has nonspecific indirect effects on many unrelated epitopes. The advent of powerful DNA sequencing techniques, collectively known as next generation sequencing (NGS), drastically changed the ability to screen yeast selection output, allowing parallel sequencing of large cell populations within different windows of antibody reactivity. Massive analysis allows us to exclude mutations expected to result in global structure changes and compare the prevalence of the remaining single mutations among the unselected yeast population and the cells selected by each antibody. Those original residues highly enriched among antibody-reactive yeasts are considered to belong to the functional epitope [65].

Combining NGS screening with rationally designed single-mutated yeast display libraries guarantees maximal usefulness of the explored sequence space [66,67]. This kind of library only contains replacements at positions where side chains are solvent-exposed (and likely to interact with other molecules), while preserving the inner core to maintain global antigen structure. Careful selection of the positions to be mutated is accompanied by the inclusion of most amino acid substitutions at each one to obtain a nearly comprehensive assessment of side chains’ contribution to epitope formation. Cys, Pro and Gly are excluded as substituents, as the first one can cause structural changes due to the formation of non-natural disulfide bonds, and the two others can distort the backbone. Recent advances in epitope mapping by yeast display have taken advantage of a very efficient way of constructing single-pot saturation mutagenesis libraries containing all the single-mutated alanine replacement variants derived from a given antigen [68,69,70,71]. Yeast display has been extremely useful to delineate the whole landscape of potential escape mutations of SARS-CoV-2 emerging receptor binding domain (RBD) variants [21,22,72,73,74,75].

An interesting modality of epitope mapping based on yeast display, despite technical similarities to functional mapping, could be better considered as a structural mapping approach. While alanine replacement libraries are useful to assess the contribution of side chains replaced by the short Ala moiety, and randomization libraries reveal the physico-chemical properties ‘preferred’ by the antibody at each position, libraries made of Cys replacement-containing molecules provide a simple way to label each targeted position with a bulky group through the highly reactive sulfhydryl moiety [76]. When a residue in close neighborhood to the antibody paratope in the complex is modified (even if it does not contribute to binding), recognition is sterically hindered. This method is thus an effective way of identifying all the residues that are in the close vicinity of the antibody, which form the structural epitope.

**Figure 3:**
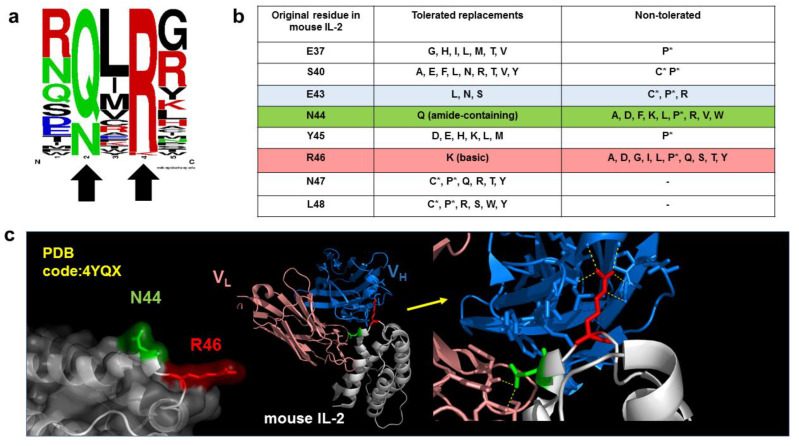
Identification of an epitope in mouse Interleukin-2 by different methods (**a**) The weblogo represents the common peptide motif identified in 45 out of 47 peptide ligands selected from a random phage-displayed decapeptide library with the antibody JES6-1A12 (anti-mouse Interleukin-2). The recurrent motif (Q/N)X(R/K) is distinguished by the presence of an amide-containing residue (Q/N, represented in green) and a basic amino acid (R, occasionally replaced by K, red), which are indicated with arrows [11]. The residue between them, as well as the flanking amino acids (aa), are highly variable. Letter size is proportional to the frequency of a given residue among selected peptides. (**b**) Mutagenesis scanning of phage-displayed mouse IL-2 with the same antibody revealed the importance of N44 and R46, which could only be replaced by the other amide-containing and basic amino acid, respectively [11]. E43 is a minor contributor, as shown by the existence of tolerated and nontolerated mutations at this position. The other residues could be substituted by different aa with diverse properties without affecting antibody recognition. In some cases, replacements by Cys and Pro were not tolerated, probably due to their effects on global protein structure. (**c**) Crystal structure of the antigenic region that contains N44 and R46 (left), and its interface with JES6-1A12 variable domains (center) [77]. The zoom-in view (right) shows the interactions of the two already identified critical aa (N44 and R46) with light and heavy chain variable regions. Dotted yellow lines indicate polar contacts established by them.

Figure 3 illustrates functional epitope mapping of an anti-Interleukin-2 antibody successfully accomplished by two different methods (identification of peptide mimotopes from a random phage-displayed library and site-directed mutagenesis of the whole phage-displayed antigen) [11]. The results from both techniques are fully compatible: the isolated peptide motif resembles in spacing and physico-chemical properties the cluster of critical residues defined by mutagenesis. This functional epitope perfectly matches with the analysis of the crystal structure of the complex, which was subsequently described in an independent study [77]. This case is an example of the power of combinatorial biology methods to delineate precise maps of target epitopes.

## 6. Combinatorial Biology Methods Reveal Singularities in the Chemistry of Epitopes

The tolerance profile to mutations varies widely from one epitope to another. While some cannot accept any change (not even a highly conservative one) at the critical positions, others are recognized after apparently more drastic changes. Complete abrogation of recognition of EGF-R by cetuximab after the conservative replacement I467M illustrates the exquisite sensitivity to epitope modification [61]. Tolerated exchange of hydrophobic residues (I, L, M, V) in other epitopes [10] implies that the presence of a hydrophobic moiety at a given position of the interface is frequently necessary and sufficient for binding. While the two negative amino acids can be exchanged in some epitopes, highlighting the importance of ionic bonds in binding, the replacements D75E, E76D and E119D totally abolish binding of mAb JES6-5H4 to mouse IL-2, reflecting the need for a perfect accommodation of these side chains into the binding pocket [10].

Epitope mapping results using mutagenesis scanning challenge our notions of conservative and nonconservative amino acid replacements. Several measures have been proposed to evaluate the differences between amino acids, based on physico-chemical distance between them, mutational distance (determined by the genetic code and mutational biases) or in evolutionary exchangeability (how often a given residue is replaced by another one in conserved protein families) [78,79,80]. Tolerability profile to mutations within functional epitopes does not adjust strictly to any of these rules. The critical attributes of each amino acid that should be kept to maintain recognition depend on the particular antibody. For instance, sometimes only tyrosine and phenylalanine residues can be exchanged without affecting antigenicity, pointing to the relevance of their almost-identical aromatic rings (differing only by a hydroxyl group in the former), whereas in other epitopes, tyrosine and histidine are exchangeable, reflecting that the two different rings can fulfill a similar functional role [10].

Another interesting example is related to Lys/Arg residues. Their exchangeability, for instance in the case of K443 in cetuximab epitope, is usually thought to be related to the positive charges they exhibit. Nevertheless, K465 in the same antigen can be replaced by both Arg and Leu [61]. As Leu is neutral and nonpolar, its ability to contribute to epitope formation can be interpreted as an indication that the critical features at this position are not related to charge, but to hydrophobic interactions established by the aliphatic chains also present in Lys and Arg.

## 7. Exploring the Other Side of the Interaction: Functional Paratope Mapping

Functional recombinant versions of the antibody binding sites can be displayed on phage and yeast, either as single chain Fv fragments comprising only the two variable domains of heavy and light chains connected by a linker peptide, or as Fab fragments assembled by covalent linkage of two polypeptides (the light chain and a second recombinant protein including heavy chain variable region and CH1 constant domain) [61,62]. As the binding site is composed by two different polypeptides, paratope exploration can start with the assessment of the contribution of each one to binding. This can be done through a technique called chain shuffling: either heavy or light chain is kept invariant, while the other chain is replaced by a diverse collection of light or heavy chains [81]. Some paratopes keep full antigen-recognition capacity despite replacement of one of the chains (usually the light chain) by multiple surrogate ones [82]. This result shows the predominant role of the other chain (frequently the heavy chain) in paratope function. Recognition might also depend on a significant contribution of both chains.

The involvement of residues within the hypervariable loops of one or both variable regions can be further explored by combinatorial mutagenesis [61,83,84,85]. CDR sequences or selected aa within them can be fully randomized, soft-randomized, replaced by Ala or by residues closely related to the original ones (homolog scanning), giving rise to antibody libraries. Diversification is followed by selection of antibody fragments keeping antigen reactivity. Comparison of the frequency of the original residues in the unselected library and among antigen-selected fragments reveals to what extent CDRs can be modified without losing binding. The output is the identification of those critical resides that cannot be changed, other aa that need to keep certain physico-chemical features to support recognition, and also positions that, despite belonging to the CDRs, are irrelevant for antigen binding. Methods that introduce a limited degree of diversity in the paratope, such as soft-randomization, are ideal for this purpose. Massive introduction of modifications could result in the recognition of a different epitope or in a large rearrangement of the paratope that creates a new binding mode, reducing the usefulness of this approach. The number and location of functionally important residues can vary from one paratope to another, although the third hypervariable regions (particularly those of the heavy chains) tend to play a significant role [61,85].

Library screening results can be confirmed by site-directed mutagenesis to segregate the effects of individual replacements. The availability of detailed functional information about the contribution of every residue in the paratope and in the epitope can be a solid starting point to guide in silico generation of binding models, providing pictures of antibody–antigen complexes that are reluctant to crystallization. Multiple theoretical solutions tend to arise from computer-mediated molecular docking studies, and choosing the ones that are compatible with functional landscapes of both epitopes and paratopes is an effective way to filter reliable models [61].

**Figure 4:**
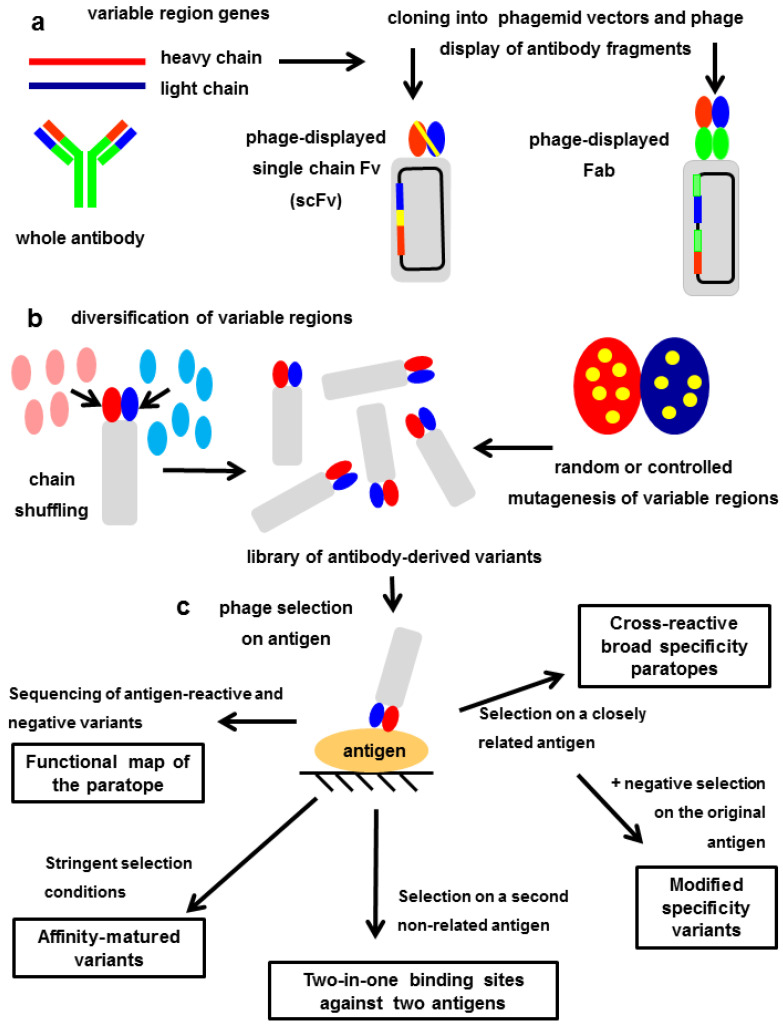
Phage display, characterization and directed evolution of antibody fragments (**a**) Cloning of antibody variable regions and display of functional scFv and Fab antibody fragments. (**b**) Large libraries of phage-displayed antibody fragments are obtained by replacing either heavy or light chain variable regions with diverse collections of homologous variable regions (chain shuffling), or by introducing random/controlled mutations within them. (**c**) Phage panning on an immobilized antigen leads to segregation of variants keeping antigen reactivity and negative variants. The analysis of their sequence profiles reveals those critical residues required for recognition, and delineates a functional map of the paratope. Alternative selection strategies can result in fine-tuning of binding properties. Stringent selection conditions allow the isolation of antibody fragments with increased target affinity (in vitro affinity maturation). Selection on a second antigen structurally related to the original one gives rise to either cross-reactive binders with broader specificity, or new binding sites with a subtle specificity change (if variants recognizing the original antigen are excluded during panning). Selection on a second nonrelated antigen can result in the generation of new binding sites with dual specificity.

Figure 4 shows the workflow to display and modify antibody fragments in a phage-based platform. Depending on the selection strategy, the final output can be functional mapping of the paratope (see above), or fine-tuning of the binding properties (affinity and specificity). The next three sections describe directed evolution of new binding sites.

## 8. In Vitro Affinity Maturation: The Challenge of Increasing the Interaction Strength without Losing Fine Specificity

The basic techniques involved in affinity maturation using combinatorial biology methods are the same described in the previous section for paratope exploration: display of functional antibody fragments, diversification of their sequences (by chain shuffling, randomization and/or controlled mutagenesis) and antigen-driven selection of binders. However, the ultimate goal determines critical differences in the experimental workflow. Comprehensive functional exploration of a given paratope benefits from the characterization of a broad range of variants keeping epitope specificity and affinity, while in vitro affinity maturation is aimed at focusing in a few variants with the maximal attainable binding strength to the same epitope. Therefore, paratope mapping often arises from a single selection round on abundant antigen, to maximize diversity and avoid interclonal competition for binding. The idea behind affinity maturation-oriented panning is increasing selective pressure to pick those antibody variants able to win the competition. Multiple enrichment rounds, limiting amounts of the antigen, stringent washes, and the presence of soluble antibody as competitor, are used to reach that goal [60,62,86]. Both phage- and yeast-displayed antibody libraries are suitable for in vitro affinity maturation [87,88].

The modular nature of paratopes, formed by heavy and light chain variable regions, is exploited in two ways during affinity maturation. Light-chain shuffling is a method of diversification commonly used to obtain stronger binders, while keeping heavy chains invariant [89,90]. Alternatively, diversification of both variable regions, either by shuffling or by mutagenesis, can be attempted in separate libraries. The combination of independently selected heavy and light chain variable regions, having individual contributions to affinity increases, can result in further binding improvements due to additive/cooperative effects when they are assembled in a single molecule [60,86]. Mutations in different CDR loops can also be selected in parallel from several libraries and subsequently combined.

The desired increase in the binding strength arises from the enhancement of already existing interactions of the original paratope, as well as from the creation on new bonds with the antigen. The reshaping of the interaction network, however, should not be too extensive, because keeping fine specificity of the parental antibody is often a pre-requisite. This can be guaranteed a priori by carefully controlling the number and identity of simultaneously targeted positions and their diversification level. Additionally, residues, clusters of aa, or even whole variable domains (in the case of chain shuffling) already identified to be critical for antibody function could be conserved to preserve crucial interactions with the epitope, just reinforced by new contacts arising during affinity maturation. Conservation of fine epitope specificity can be verified a posteriori through the application of functional mapping methods described in the previous sections to both the parental antibody and the affinity-improved variants [62].

Even if no gross epitope shift is observed, the affinity increase by itself could result in previously undetectable cross-reactivity with chemically related epitopes on similar antigens, leading to undesired off-target side effects during antibody therapy. Another risk is that the enhancement in affinity is associated with the generation of ‘sticky’ paratopes that could exhibit promiscuous nonspecific binding to multiple molecules [91]. The use of potent blocking agents such as skim milk and stringent washes to exclude any nonspecific binder during library selection, as well as the inclusion of additional steps of negative selection/depletion that could minimize the risk of picking cross-reactive binders, increasing the chances of successful affinity maturation. The latter can be performed by pre-panning on a second structurally related antigen (not intended to be recognized), by using it as a soluble competitor during phage panning on the nominal antigen, and by screening binders against both antigens to discard the cross-reactive ones [86].

There are no rules about the magnitude of affinity increases that should be expected. Even though in vitro affinity maturation methods are powerful enough to reach binding improvements of several orders in the most impressive examples [92,93,94,95], sometimes small increases (less than 10-fold) are technically challenging [62]. On the other hand, the ideal affinity for practical applications is not always the highest one. Minimal changes can already have an impact in antibody biological functions and analytical applications [96], and frequently there seems to be an optimal affinity window for therapeutic effects. This optimal range often can be theoretically predicted [97] but can also be explored experimentally in animal models [98,99], and it is influenced by aspects such as the balance between therapeutic efficacy and toxicity [100], and the limitations in bioavailability of high affinity antibodies that can be sequestered at tissues exhibiting low antigen expression or at peripheral areas of the target organ (or tumor) [101].

## 9. Modulating the Original Antibody Specificity towards Closely Related Antigens

Cross-reactivity or fine specificity shift, undesired outcomes in most affinity maturation procedures, can be envisaged as desirable results in some particular cases. Once antibodies against poorly immunogenic antigens or infrequently recognized epitopes are available, they can be used as starting points to engineer antibody versions that recognize structurally related epitopes on similar antigens. This can be accomplished through controlled diversification of CDRs of the starting antibody and selection on the second target. Depending on the goal, cross-reactive binders keeping the ability to recognize the original target epitope can be excluded or not during selection and screening. Selection from such biased libraries that explore a narrow sequence space focused on recognition of a certain type of antigen(s), instead of a universal combinatorial antibody repertoire made of broadly diverse heavy and light chains, should have advantages to isolate difficult-to-obtain antibodies.

**Figure 5:**
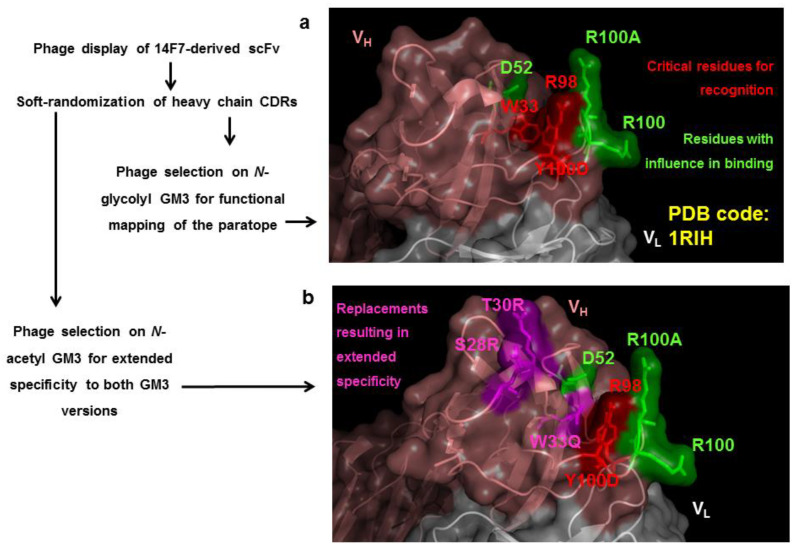
Directed evolution of cross-reactive paratopes from an anti-ganglioside antibody. (**a**) Functional map of 14F7 antibody paratope, defined through selection of phage-displayed antibody fragments from a heavy chain CDRs soft-randomization library on immobilized *N*-glycolyl GM3 ganglioside antigen [85]. Residues W33, R98 and Y100D were strictly conserved among antigen-reactive variants (critical determinants of antibody recognition, highlighted in red), while D52, R100 and R100A (indicated in green) also played a role in binding. (**b**) Selection on a second antigen (the structurally related *N*-acetyl GM3) rendered cross-reactive binders with broader specificity for both gangliosides. The newly evolved paratopes kept the same functionally relevant residues, except for three changes that determined specificity extension. Modifications arising during directed evolution included the replacements W33Q, S28R and T30R (magenta). The overlapping between the original paratope and the cross-reactive variant indicates that they share a similar binding mode with subtle differences, even in the absence of structural information about the complexes.

This approach has been used to obtain new antibodies against different members of the families of steroids [102] and sulfonamides [103], which exhibit subtle chemical differences in their structure, and to expand the reactivity of a unique high-affinity antibody previously obtained against the *N*-glycolyl GM3 ganglioside towards another tumor-associated ganglioside, the poorly immunogenic *N*-acetylated GM3 version [85]. Figure 5 shows the overlapping between the functional maps of the original binding site and the cross-reactive paratope obtained in the latter example. This case illustrates how in vitro evolution of desired cross-reactivities can provide theoretically unpredictable solutions, in the absence of previous structural knowledge about the antibody-antigen complex. Remarkably, CDR mutations arising upon selection on the second antigen, more hydrophobic than the original one, involved the appearance of polar residues, which was surprising.

In vitro cross-reactivity evolution, affinity maturation, and the combination of both have been used to obtain broadly neutralizing antibodies against complex families of related toxins and venoms [104,105,106]. Other application involving cross-reactivity engineering is the generation of antibodies against human targets with the ability to recognize the equivalent (although not identical) epitope present in the homologous antigens of other species [107,108]. These tools would add value to proof-of-concept experiments in animal models, performed with antibodies that are very similar to the therapeutic version intended to be used in humans.

## 10. Totally Divergent Specificities in A Single Binding Site: Two-in-One Paratopes

A different approach to specificity engineering is related to the accommodation of two structurally unrelated epitopes in the same paratope. Such a change, known to be chemically possible since early studies of antibody recognition of unrelated peptide ligands (see Section 4), would result in molecules targeting two epitopes of therapeutic interest in two different antigens, simplifying the production of multi-purpose two-in-one single agents. Display of a first functional binding site, followed by controlled diversification aimed at keeping the original binding properties to the nominal antigen and selection on the second antigen, can produce modified paratopes able to accommodate both antigens. Subsequent affinity maturation and specificity optimization might be required to reach the desired binding properties. The concept of dual specificity within a single paratope, first shown with antibodies able to target HER-2 and vascular endothelial growth factor (VEGF) [109,110], was further expanded to other antigen pairs using diverse protocols [111,112,113,114]. It was possible to dissect the differential involvement of CDR loops and residues in recognition of each antigen at high resolution [113]. The existence of such engineered dual-specificity paratopes poses the question of whether they can also evolve in nature to target two different epitopes that appear in the organism during an antigenic challenge such as an infection. The fact that this kind of recognition is possible does not necessarily mean that it is biologically relevant.

A very smart strategy to engineer dual-targeting antibody fragments (called DutaFabs) in an expedited way relies on the a priori division of the antibody binding sites, formed by a given pair of heavy and light chain variable regions, into two sides that can be considered spatially separated and independent functional paratopes. The so-called H-side comprises heavy chain CDRs 1 and 3 and light chain CDR2. The L-side paratope encompasses light chain CDRs 1 and 3, together with heavy chain CDR2. The rationale for such a division was based on geometrical considerations about the architecture of binding sites. Then, two independent antibody libraries are constructed on the scaffold formed by this combination of variable domains. Only the residues belonging to the hypervariable loops of one side are diversified in each of them. Selection from each library on a single target antigen is supposed to render binders whose recognition ability resides in the side that was diversified in the starting library. If antibody fragments against two different antigens are selected from the two libraries, their functionally relevant loops do not overlap and could in principle be combined in a single binding site that should interact with both antigens. The concept, elegantly proved in the case of a DutaFab able to bind simultaneously VEGF-A and Platelet-derived growth factor (PDGF-BB) [115], could eventually face practical limitations due to steric hindrance effects mediated by loops located in one paratope side on recognition by loops belonging to the second side. Nevertheless, this approach is one of the most creative innovations in the very competitive field of antibody engineering during the last few years.

## 11. General Remarks and Future Prospects

The usefulness of display technologies to explore fine epitope specificity of antibodies and to modify their binding properties has been validated by decades of successful application and by thousands of publications. During such a long time, however, technological platforms have significantly evolved. Basic library construction and high-throughput screening procedures are common research tools that can be used in a conventional molecular biology laboratory having the required expertise and minimal material resources. Nevertheless, industrial exploitation has been linked to further increases in the scale of molecule discovery, characterization and optimization, based on miniaturization and automation of every step.

One of the major technological breakthroughs that has contributed to shape the current landscape of library generation and screening is the emergence of NGS. While limited sequence sampling (using the Sanger method) provided a gross idea of library variability from the very beginning of display technologies, deep DNA sequencing of antibody and antigen repertoires through NGS techniques has revealed the actual diversity, completeness, redundancy and biases within these collections, helping to improve their design. Furthermore, NGS analysis of selected multi-clonal populations is powerful enough to underscore a plethora of significant selection-driven molecular patterns beyond the few molecules that typically dominate the enrichment procedure and can be identified through limited clonal screening. Deep sequencing of libraries and library products is becoming a common practice in many laboratories [65,66,67,68,69,70,71,72,73,74,116].

Despite the success of the most extended display technologies (phage, yeast and to a lesser extent bacterial display), biosynthesis of proteins such as antibodies and human antigens in any of these systems occurs in a non-natural host environment, where post-translational modifications (for instance, glycosylation) are absent or chemically different from those introduced by mammalian cells. Additionally, folding pathways in host cells lacking the whole mammalian biosynthesis/secretion machinery can lead to improperly folded products. Such concerns have promoted efforts aimed at developing efficient mammalian display systems, where a single recombinant protein is anchored at the membrane of a cell that can be selected by FACS depending on the binding properties of the displayed molecule [117]. Proteins modified and selected in that way reproduce structural and functional features of their natural counterparts to a greater extent, being better tools to characterize the original interactions. Therapeutic molecules derived from mammalian display should be ideally suited for subsequent industrial production in mammalian host cells such as CHO and HEK-293.

Another future-oriented research area is epitope-guided antibody discovery, aimed at producing antibodies with fine specificities defined a priori. This can be accomplished by using small target molecules reproducing the epitope of interest for antibody generation. A second possibility is rational design of CDRs able to interact with the chosen epitope. Such theoretical design can take advantage of prior knowledge about the interactions of epitopes closely related to the one intended to target [118,119]. On the other hand, de novo design is based on entirely in silico screening of sequence diversity within the scaffold of known canonical CDR conformations derived from antibody structural databases [120,121,122,123]. A third option is molecular grafting of specificity determinants from a non-antibody protein or peptide known to interact with the target into an antibody framework [124,125,126,127].

Despite moderate advances in the field, the creation of antibodies with pre-determined fine specificities is not very extended, and the current scenario is dominated by immunization procedures and antibody library screening, which give rise to antibodies against multiple epitopes. Designed antibodies often do not reach the affinity levels obtained through other techniques, and they require further optimization. Therefore, efficient combinatorial approaches for epitope mapping and antibody-directed evolution will still be required during the next years. In a longer term, all the lessons about the chemistry of antibody-antigen reactions that the application of these methods is teaching us could pave the way for ‘intelligent’ design of antibodies with pre-defined specificities and affinities.

## Data Availability

No new data were created or analyzed in this study. Data sharing is not applicable to this article.
